# Borocarbonitride Layers on Titanium Dioxide Nanoribbons for Efficient Photoelectrocatalytic Water Splitting

**DOI:** 10.3390/ma14195490

**Published:** 2021-09-23

**Authors:** Nuria Jiménez-Arévalo, Eduardo Flores, Alessio Giampietri, Marco Sbroscia, Maria Grazia Betti, Carlo Mariani, José R. Ares, Isabel J. Ferrer, Fabrice Leardini

**Affiliations:** 1 Departamento de Física de Materiales, Campus de Cantoblanco, Universidad Autónoma de Madrid, E-28049 Madrid, Spain; joser.ares@uam.es (J.R.A.); isabel.j.ferrer@uam.es (I.J.F.); fabrice.leardini@uam.es (F.L.); 2 Centro de Nanociencias y Nanotecnología (CNyN), Universidad Nacional Autónoma de México (UNAM), Ensenada 22860, BC, Mexico; eduardoe.floresc@gmail.com; 3 Dipartimento di Fisica, Università di Roma ‘La Sapienza’, I-00185 Rome, Italy; alessio.giampietri@uniroma1.it (A.G.); marco.sbroscia@uniroma1.it (M.S.); maria.grazia.betti@roma1.infn.it (M.G.B.); carlo.mariani@uniroma1.it (C.M.); 4 Instituto Nicolás Cabrera, Campus de Cantoblanco, Universidad Autónoma de Madrid, E-28049 Madrid, Spain

**Keywords:** borocarbonitride, TiO_2_-BCN heterostructures, water splitting, photoelectrocatalysis, X-ray photoelectron spectroscopy, graphene analogues, hybrid structures

## Abstract

Heterostructures formed by ultrathin borocarbonitride (BCN) layers grown on TiO_2_ nanoribbons were investigated as photoanodes for photoelectrochemical water splitting. TiO_2_ nanoribbons were obtained by thermal oxidation of TiS_3_ samples. Then, BCN layers were successfully grown by plasma enhanced chemical vapour deposition. The structure and the chemical composition of the starting TiS_3_, the TiO_2_ nanoribbons and the TiO_2_-BCN heterostructures were investigated by Raman spectroscopy, X-ray diffraction and X-ray photoelectron spectroscopy. Diffuse reflectance measurements showed a change in the gap from 0.94 eV (TiS_3_) to 3.3 eV (TiO_2_) after the thermal annealing of the starting material. Morphological characterizations, such as scanning electron microscopy and optical microscopy, show that the morphology of the samples was not affected by the change in the structure and composition. The obtained TiO_2_-BCN heterostructures were measured in a photoelectrochemical cell, showing an enhanced density of current under dark conditions and higher photocurrents when compared with TiO_2_. Finally, using electrochemical impedance spectroscopy, the flat band potential was determined to be equal in both TiO_2_ and TiO_2_-BCN samples, whereas the product of the dielectric constant and the density of donors was higher for TiO_2_-BCN.

## 1. Introduction

The current energetic model based on fossil fuels is unsustainable from an environmental perspective, as it is one of the leading causes of global warming and climate change [1,2,3]. The focus is now placed on solar and wind energy, which have the problem of being intermittent, which points to the necessity of developing new ways of storing energy. 

Among all the energy storage methods, energy storage using molecular bonding stands out, such as the one in the hydrogen molecule. Hydrogen has been reported to be a suitable energy vector and a clean energy fuel if its production comes from renewable sources [2,4].

In 1972, Honda and Fujishima reported a way to obtain hydrogen by carrying out a photoassisted water splitting reaction using TiO_2_ as the photoanode [5]. Since then, this effect has been considered one of the cleanest methods to obtain green hydrogen and a promising strategy to overcome the environmental and energy crises [6,7]. The water-splitting reaction consists of two partial reactions, the oxygen evolution reaction (OER) and the hydrogen evolution reaction (HER). The OER is the rate-determining step as it involves the transfer of four electrons [8], and this is the reaction we will tackle in this paper.

The low cost, stability and non-toxicity of TiO_2_, as well as its adequacy to carry out the water splitting reaction, has drawn the attention of many groups who have reported the good properties of this material by synthesizing it in different structures and nanostructures [9,10].

To increase the charge transfer between the electrode and the electrolyte, metals nanoparticles, such as Pt and Ni, are commonly used as active electrocatalytic sites for water splitting [11]. The main focus is now placed on developing new metal-free compounds to be used as catalysts for the oxygen and hydrogen evolution reactions. 

Graphene analogues and other 2D materials have demonstrated to be highly interesting metal-free compounds with a wide range of applications in electrocatalysis [12]. These layers have the advantage of being distributed along all the electrode surface, increasing the reaction area in comparison to the metal nanoparticles. Among these 2D materials borocarbonitride compounds (BCNs hereafter) stand out, which are low cost and highly stable materials formed by h-BN and graphite domains. BCNs have been reported to be efficient electrocatalysts for the HER [13,14] and, most interestingly, have recently been proved to be an efficient electrocatalysts for the OER, improving the properties of TiO_x_ substrates for this reaction [15].

In this article we have first confirmed the utility and versatility of plasma enhanced chemical vapor deposition to grow BCN on samples with different morphologies. This technique has been previously used to grow BCN on TiO_x_, Cu, and other flat substrates [15,16]. In this work, BCN was grown, for the first time, on nanostructured TiO_2_ samples. In particular, BCN was grown on TiO_2_ nanoribbons, obtained by the thermal annealing of TiS_3_ [17]. A deep characterization of TiO_2_ nanoribbons, with and without BCN, as well as the starting material, TiS_3_, was made with scanning electron microscopy (SEM), X-ray diffraction (XRD), Raman spectroscopy and diffuse reflectance measurements. X-ray photoelectron spectroscopy (XPS) characterization of bare and BCN-covered TiO_2_ nanoribbons was performed.

Finally, we demonstrated the good properties of the BCN as an electrocatalyst of the OER by improving the charge transfer between the TiO_2_ nanoribbons electrode and the KOH aqueous electrolyte, under dark and light conditions. 

## 2. Materials and Methods

### 2.1. Synthesis

The starting TiS_3_ material has been obtained by the sulfuration of Ti disks (15 mm diameter, Good Fellow 99.5%) in sealed Pyrex ampoules for 20 h at 550 °C, using sulfur powder as sulfur source [18,19].

TiS_3_ nanoribbons were squashed in one direction and oxidized on a hot plate at 300 °C in air (the decomposition temperature of TiS_3_ [17]) for 20–30 s, which allowed us to obtain the desired TiO_2_ nanoribbons. Figure S1 shows the change in the color of the samples, from black to white, at a glance. 

BCN was grown on TiO_2_ using PE-CVD to get the TiO_2_-BCN heterostructures. The TiO_2_ sample and a single-source molecular precursor (methylamine-borane, BH_3_NH_2_CH_3_) were placed inside a Pyrex ampoule immersed in liquid nitrogen and then sealed under vacuum at a pressure around 10^−5^ mbar. When the sealed ampoule reaches room temperature, the molecular precursor is in equilibrium with its vapor pressure in the 10^−2^ mbar range. Then a plasma was activated inside the ampoule using the radiation of a conventional microwave oven. More details about this technique can be found elsewhere [15,16].

### 2.2. Characterization

The starting material, TiS_3_, as well as TiO_2_ and TiO_2_-BCN heterostructures, were characterized by using different techniques.

The morphology of the samples was investigated by scanning electron microscopy (SEM) using a Hitachi S3000 instrument. Additionally, they were characterized using Raman spectroscopy with a WITec ALPHA 300AR instrument using a confocal microscope with lenses of 20× and 100×. The used laser had a power of 0.2 mW and an excitation wavelength of 532.3 nm.

The structural properties were measured using a Panalytical X’Pert Pro X-ray diffractometer at glancing angle configuration (incident angle of 1.7°, CuKα radiation).

The optical reflectance spectra were recorded in a UV/VIS/NIR Perkin-Elmer LAMBDA 950 spectrophotometer equipped with an integrating sphere to collect the reflecting flux, using a spot size of 21 mm^2^ in the 300–2000 nm spectral range.

The composition on the surface of TiO_2_ and TiO_2_-BCN samples was investigated by X-ray photoelectron spectroscopy (XPS). These measurements have been carried out in an ultrahigh-vacuum (UHV) chamber, with a base pressure in the low 10^−10^ mbar range. Photoelectrons excited by an Al Kα photon source (hυ=1486.7 eV), were measured by a hemispherical electron analyzer (VG Microtech Clam-2) in a pass energy mode set at 50 eV for Ti and C, and 100 eV for B and N. Further details about the procedure are available in [15,16,20,21]. The binding energy (BE) was calibrated by acquiring the Au4f_7/2_ (84.0 eV of BE) core-level after each measurement. The measurements were done after annealing the samples at 320 °C for 1 h, at 110 °C for 15 h and 320 °C for another hour in UHV.

Electrochemical measurements were done in a three-electrode cell. Our material, which had an apparent area of 1.3 cm^2^, was placed as the working electrode (WE), a platinum sheet (9 cm^2^) as the counter electrode (CE), and, as the reference electrode (RE), an Ag/AgCl electrode filled with 1M KNO_3_ (XR440 from Radiometer Analytical) was used. Its electrode potential was 484 mV vs. a normal hydrogen electrode (NHE). These three electrodes were immersed in 0.1 M (pH = 13.0) and 1.0 M (pH = 13.7) KOH aqueous electrolyte. The potentials are converted to the reversible hydrogen electrode (RHE) using Equations (1) and (2):

(1) $$E_{NHE}=E_{Ag/AgCl}+E_{Ag/AgCl}^{0}$$

(2) $$E_{RHE}=E_{NHE}+0.059\cdot pH$$

where $E_{NHE}$ is the electrode potential in the *NHE* scale, $E_{Ag/AgCl}$ is the experimental electrode potential measured vs. *Ag*/*AgCl* reference electrode, $E_{Ag/AgCl}^{0}$ is the electrode potential vs. the *NHE*, $E_{RHE}$ is the electrode potential in the RHE scale, and pH is the pH of the solution used.

The electrochemical and photoelectrochemical (PEC) measurements were done using a potentiostat–galvanostat PGTAT302N (Autolab) provided with an integrated impedance module, FRAII. The WE was illuminated with a Xe lamp (Jobin Yvon) of 75 W in the visible-UV range. The maximum light intensity reaching the sample was 140 mW. During electrochemical measurements, argon was bubbled at a constant flow of 20.0 sccm. To characterize the electrodes under both dark and illumination conditions, linear sweep voltammetry (LSV), cyclic voltammetry (CV) and current density measurements at a fixed potential were employed. In addition, electrochemical impedance spectroscopy (EIS) was used to characterize the interface electrolyte-semiconductor, by using a sinusoidal AC voltage signal with an amplitude of 10 mV and a variable frequency between 100–1000 Hz. 

More details about the photoelectrochemical experimental system can be found in the Supplementary Materials (Figure S2).

## 3. Results and Discussion

### 3.1. Morphological and Structural Characterization

**Figure 1:**
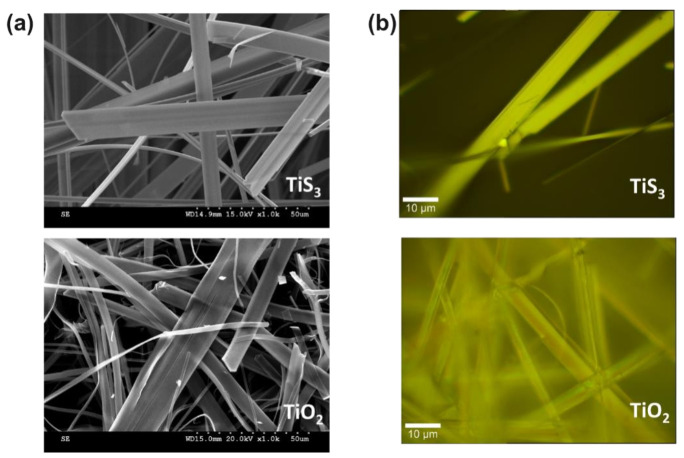
(**a**) Scanning electron micrographs of TiS_3_ (top) and TiO_2_ (bottom). (**b**) Optical microscopy image of TiS_3_ (top) and TiO_2_ (bottom).

The oxidation of the TiS_3_ nanoribbons was performed to change the atomic structure and the composition of the material, without changing the morphology of the sample, in order to obtain TiO_2_ nanoribbons. Figure 1a reports the morphology of TiS_3_ before and after the oxidation. It is clear from the SEM measurements, that there was no significant change in the morphology, apart from a slight increase of the roughness on the TiO_2_ surface compared to TiS_3_. We also acquired optical microscopy images to have a deeper understanding of the change on the surface of these nanoribbons (Figure 1b), which allowed us to determine that the material became more transparent to the optical microscope, in good agreement with the results reported by Ghasemi et al. [17] and with our optical characterizations shown below (see Figure 4).

**Figure 2:**
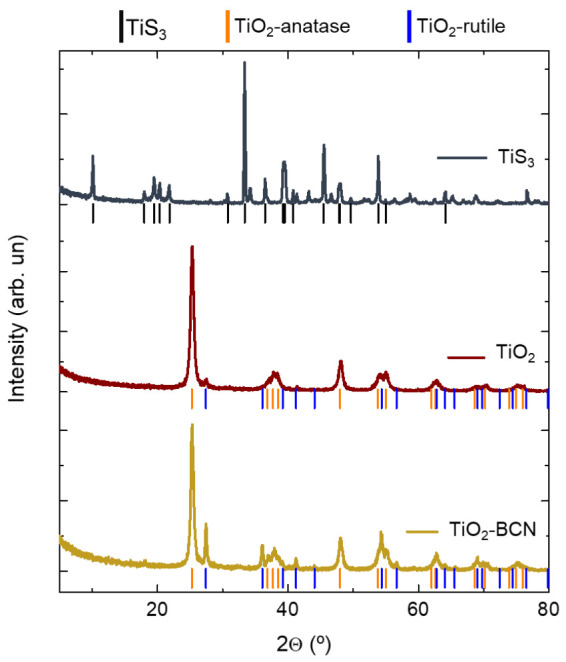
X-ray diffraction patterns for TiS_3_, TiO_2_ and TiO_2_-BCN samples. TiS_3_ peaks correspond to JDPDF 00-015-0783, TiO_2_-anatase peaks correspond to JDPDF 01-071-1167, and TiO_2_-rutile peaks correspond to JDPDF 01-073-2224.

Structural changes were also monitored via XRD after the oxidation and after growing the BCN on top of this oxidized material (Figure 2). There were clear changes in the structure due to the thermal annealing, as there was a transition from monoclinic TiS_3_ to tetragonal TiO_2_-anatase with some peaks of tetragonal TiO_2_-rutile. 

After the BCN synthesis, the intensity of the rutile peaks increased. This was ascribed to the high temperature that the sample achieved during exposure to the plasma, which modified the structure of the bulk material by crystallizing the sample from anatase TiO_2_ to rutile TiO_2_. 

**Figure 3:**
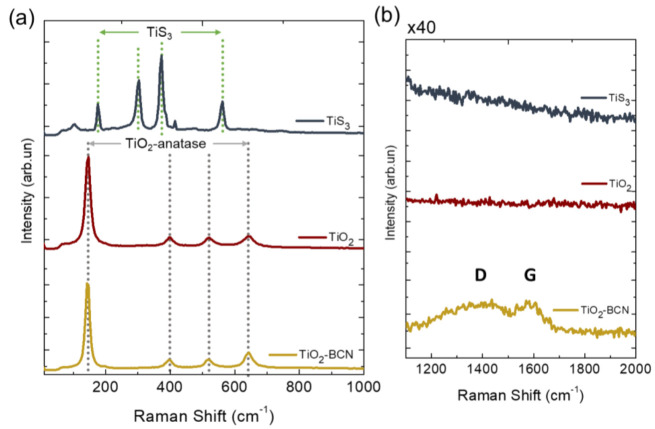
Raman spectra for TiS_3_, TiO_2_ and TiO_2_-BCN samples in the (**a**) 100–1000 cm^−1^ Raman shift range and (**b**) 1100–2000 cm^−1^ Raman shift range with a ×40 zoom in the intensity.

Raman spectra of TiS_3_ and TiO_2_ nanoribbons, reported in Figure 3, show that there is a complete change in their structure from TiS_3_ (175 cm^−1^, 303 cm^−1^, 373 cm^−1^, 562 cm^−1^) [22] to TiO_2_-anatase (145 cm^−1^, 396 cm^−1^, 518 cm^−1^, 643 cm^−1^) [10,23,24]. In the case of TiO_2_-BCN, there was no change in the Raman spectra of the nanoribbons, thus they maintained their anatase composition. The growth of the BCN in this sample was confirmed by the presence of D (1375 cm^−1^) and G (1590 cm^−1^) Raman bands, ascribed to the BCN layers graphene-like sp2 structure [15,16]. 

It must be mentioned that in the TiO_2_-BCN sample, some TiO_2_ rutile nanoribbons [25,26] were also found in the Raman spectra (Figure S3). This result is in good agreement with the increase in the rutile peaks in the XRD diffractogram for this sample. The nanoribbons were found at the borders of the samples and did not contribute to the electrochemistry as they were not exposed to the electrolyte due to the shape of the electrode holder.

### 3.2. Optical Characterization

Diffuse reflectance measurements were done using an integrating sphere to characterize the change in the energy bandgap. The reflectance (*R*) data were converted into a Kubelka–Munk function, *F*(*R*) [27] (Equation (3)), which was proportional to the optical density in the optical absorption measurements.

(3) $$F\left(R\right)\;=\frac{{\left(1-R\right)}^{2}}{2R}$$

**Figure 4:**
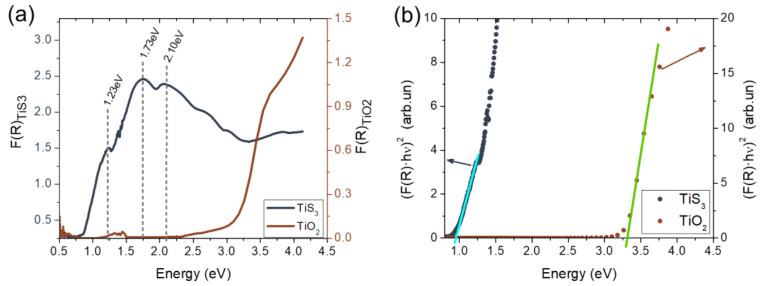
(**a**) Kubelka–Munk function obtained from diffuse reflectance measurements of TiS_3_ (left axis) and TiO_2_ (right axis). (**b**) Tauc plots of Kubelka–Munk function with the corresponding linear fit for TiS_3_ (left axis) and TiO_2_ (right axis).

Results as a function of the incident light energy can be seen in Figure 4a. At first glance, the presence of 1.23 eV, 1.73 eV and 2.1 eV absorption bands, characteristic of the TiS_3_ samples [19] can be observed, but they disappear after the oxidation process due to the transformation of TiS_3_ into TiO_2_.

To obtain the band gap energy of TiS_3_ and TiO_2_ samples, Tauc fitting equation for direct band gaps (${\left(F\left(R\right)\cdot h\nu \right)}^{2}$) has been applied to the F(R) function (Figure 4b). A clear change can be observed from 0.94±0.04 eV in TiS_3_, to 3.3±0.4 eV in TiO_2_. These results are in good agreement with the values previously obtained for TiS_3_ [19] and TiO_2_ [28].

### 3.3. Surface Chemical Composition and Element Distribution

**Figure 5:**
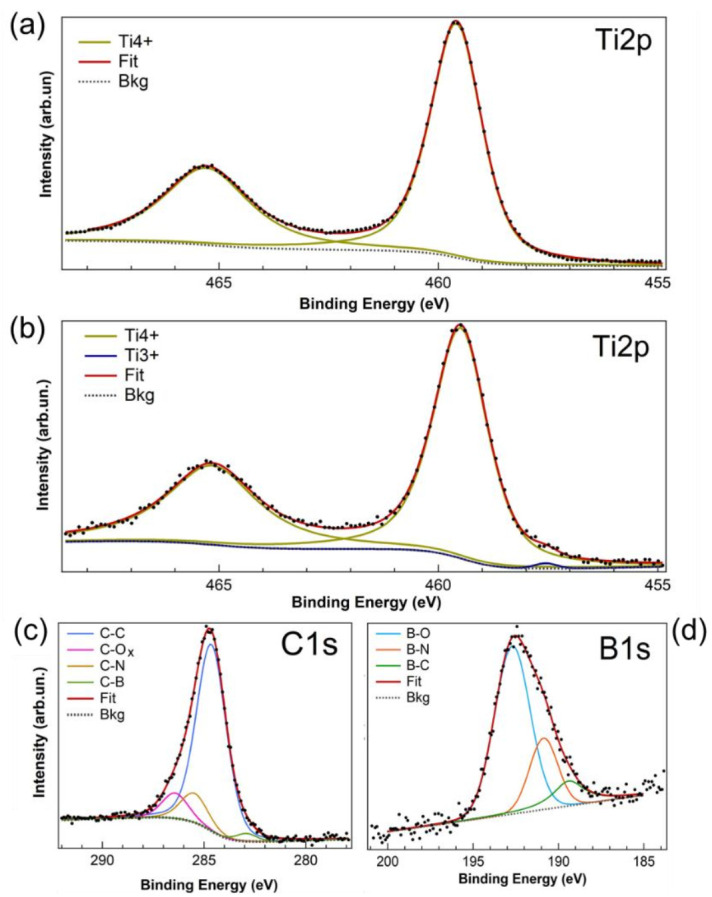
Ti2p XPS spectra for (**a**) TiO_2_ sample and (**b**) TiO_2_-BCN sample. C1s (**c**) and B1s (**d**) spectra for TiO_2_-BCN sample. Experimental data (dots), Shirley background is represented by a grey dotted line, the fitting curve for each measurement is in red, and single fitting components, as described in the legend.

For further characterization of the chemical bonding state in the surface of TiO_2_ and TiO_2_-BCN samples, XPS measurements were carried out. The Ti2p level for the TiO_2_ sample is shown in Figure 5a, and the B1s, C1s and Ti2p core levels for TiO_2_-BCN can be seen in Figure 5b–d. A fitting analysis using Voigt line shapes (combination of Gaussian and Lorentzian curves) was performed to take into account the overall experimental uncertainty and the intrinsic linewidth, respectively. A Shirley background was introduced as a fitting parameter for all the analyzed peaks. All the fitting parameters for the graphs in Figure 5 are reported in Table S1.

From this result, it is evident that the Ti2p levels show the presence of Ti^4^^+^ in both samples, which can be attributed to the formation of TiO_2_ [29]. However, in the case of TiO_2_-BCN, a small additional peak at 457.55 eV of BE appears, which is ascribed to the presence of Ti^3^^+^ bonds [30,31], probably due to small areas with defects close to the edge of the sample. Nevertheless, this component represents the 0.5% of the relative intensity, meaning that the plasma did not significantly affect the surface of the substrate for the growth times used.

In the TiO_2_-BCN sample, XPS measurements revealed that the BCN layer was composed of C and h-BN domains with high mutual doping levels of B and N in C, and of C in BN, respectively. The B1s and C1s peaks appear with a large width and are composed of more than one peak, indicating the presence of different components associated with the mutual bonding among the elements. 

The B1s core level in TiO_2_-BCN shows three peaks due to the B–C (190.51 eV), B–N (191.41 eV) [15,16,21,32,33,34] and B–O (192.96 eV) bonds [15,16,20,21,35]. The C1s core level exhibit four main components due to the C–C bonding at 284.6 eV, signature of the sp2-bonded carbon, C–N bonds (285.48 eV), C–B bonds (282.65 eV), and multiple components due to different bonds of C to O [15,16,21,32,33,34], which are unresolved and appear as a single broad band at 286.43 eV. We were not able to decompose the N1s peak in different Voigt functions, as we did with C1s and B1s peaks, since the signal of N1s is overlapped with a small signal of the Ta4p_3/2_ peak, ascribed to the tantalum clips used to fix the sample to the sample holder. The spectrum in an energy range close to the N1s peak (385–413 eV) is shown in Figure S4.

Thus, the B and C 1s core levels present the typical features associated with the mutual chemical bonds in sp2-hybridized compounds. This is a clear signature of the formation of a ternary BCN planar layer [15,16]. The amount of BCN in the sample was estimated by XPS quantification, taking into account the cross-section, obtaining a ratio between Ti and B of 7:1, which suggests that the layer of BCN was ultrathin.

### 3.4. Photoelectrocatalytic Activity for the OER

**Figure 6:**
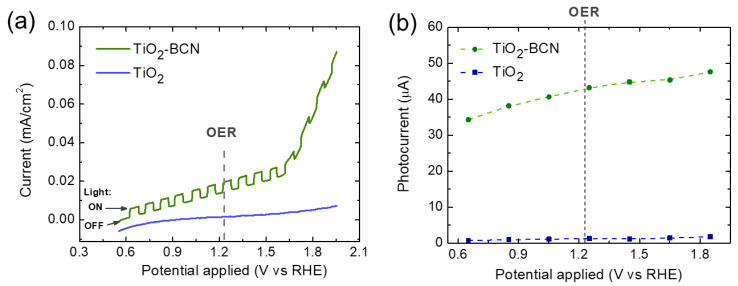
(**a**) Linear sweep voltammetry curves for bare TiO_2_ and TiO_2_-BCN under intermittent illumination conditions. (**b**) Photocurrents obtained for different constant applied potentials.

The effect of BCN on the electrocatalytic activity of TiO_2_ was investigated at first via linear sweep voltammetry (LSV) measurements under intermittent illumination conditions comparing the results from TiO_2_-BCN heterostructure and these from bare TiO_2_ one (Figure 6a). Regarding the dark condition, bare TiO_2_ presented low currents and poor catalytic activity for the OER compared to those of the TiO_2_-BCN sample, which points out the electrocatalytic effect of BCN. In fact, at the maximum applied potential (1.9 V vs. RHE), the dark current was 12-fold higher than that of the bare TiO_2_. Photocurrents were measured at different applied stationary potentials (Figure 6b), and, as can be seen, it increases from around 1 µA (bare TiO_2_) to around 40 µA (TiO_2_-BCN) at 1.23 V vs. RHE. The photocurrents were stable (Figure S5) and have a positive value, in good agreement with the nature of TiO_2_ as an n-type semiconductor [36]. It can be concluded that the BCN layer does not only improves the electrocatalytic properties of the TiO_2_ at dark conditions, but also under illumination by increasing the photocurrents. The LSV curves, as well as CV curves, were measured for different scan rates, which made it possible to determine that the current that appears in the TiO_2_-BCN sample before the OER in the LSV curve is due to the scan rate and not to another reaction (Figure S6). 

The stability has been monitored by doing series of 50 cycles at 0.05 V/s, proving the high stability of our material, as can be seen in Figure S7. Raman measurements were also made before and after the photoelectrochemical measurements to prove that there was no degradation of the sample and that the BCN layer was still there (Figure S8).

In order to characterize the interface between the electrodes (TiO_2_ and TiO_2_-BCN) and the electrolyte (KOH), EIS measurements were done. By measuring the impedance at different frequencies, the capacitance in the spatial charge region ($C_{SC}$) could be acquired, and with the Mott–Schottky equation (Equation (4)), the value of the flat band potential ($V_{fb}$) could be obtained.

(4) $$\frac{1}{C_{SC}^{2}}=\;\left(\frac{2}{\varepsilon \cdot A^{2}\cdot e\cdot N_{D}}\right)\cdot \left(V_{bias}-V_{fb}-\frac{k_{B}\cdot T}{e}\right)$$

where ε is the dielectric constant of the electrode, $\varepsilon_{0}$ is the vacuum permittivity, A is the area of the electrode, e is the charge of the electron, $N_{D}$ is the donor density, $V_{bias}$ is the applied potential, $k_{B}$ is the Boltzmann’s constant and T is the temperature. The term $\frac{k_{B}\cdot T}{e}$ can be neglected due to its low value when compared to $V_{bias}$ and $V_{fb}$.

**Figure 7:**
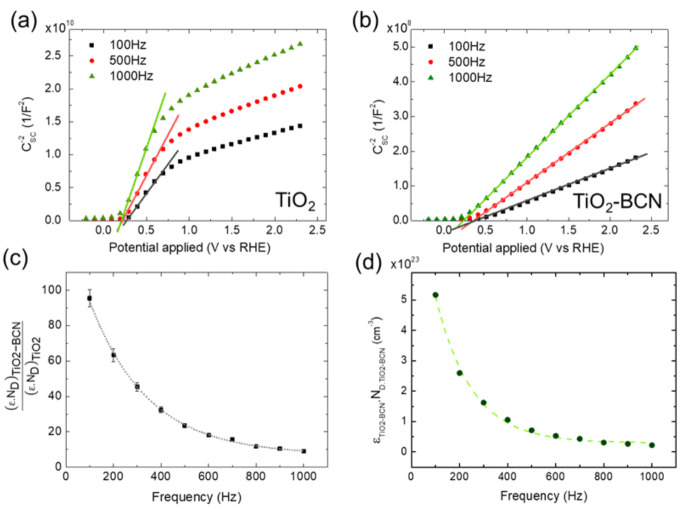
Mott–Schottky plot at three different frequencies for (**a**) sample TiO_2_ and (**b**) sample TiO_2_-BCN. (**c**) Relationship between the dielectric constant and the density of donors $(\varepsilon \cdot N_{D}$ factor) of the sample TiO_2_-BCN over that of the bare TiO_2_, as a function of the frequency. (**d**) $\varepsilon \cdot N_{D}$ factor for TiO_2_-BCN sample vs. the frequency. Fitting parameters of the exponential decay fittings represented by a grey dotted line in (**c**) and green dashed line in (**d**) are shown in Table S3.

The value of the flat band potential is highly significant in the electrochemical characterization of a semiconductor–electrolyte interface, as it is related to the bottom energy of the conduction band. Together with the bandgap energy, it is used to describe the energy levels position at the electrode–electrolyte interface, determining the adequacy of a material to carry out or not any reaction, in this case, water splitting. Figure 7a,b shows the Mott–Schottky plot at three representative frequencies for the bare TiO_2_ and TiO_2_-BCN samples. The value obtained for the flat band potential is 0.2±0.1 V vs. RHE for bare TiO_2_, and 0.2±0.1 V vs. RHE for TiO_2_-BCN. These results are in good agreement with values of the flat band potential previously obtained for TiO_2_ samples [9,10], and with a previous report about the good electrocatalytic activity of BCN for OER, in which it was shown that the BCN layer does not affect the flat band potential of the underlying material [15]. Table S2 summarizes the main differences between TiO_2_ and TiO_2_-BCN.

The slope of the Mott-Schottky fitting gives information about the dielectric constant of the material and its donor density. By using the data of the dielectric constant of TiO_2_ at 20 °C reported by Wypych et al. [37] and the slope of bare TiO_2_, its donor density was determined to be independent of the frequency and has a value of $\left(1.53\pm 0.02\right)10^{19}{\;\mathrm{cm}}^{-3}$ (Figure S9). This is in good agreement with the fact that the donor density is a characteristic parameter of the material and should not change with the frequency. However, as observed in [37], the dielectric constant is a function of the frequency. 

By comparing the slopes of TiO_2_ ($m_{{\mathrm{TiO}}_{2}})$ and TiO_2_-BCN ($m_{{\mathrm{TiO}}_{2}-\mathrm{BCN}})$ at each frequency (Equation (5)) the relationship between the product of ε and $N_{D}$ for both samples can be determined (Figure 7c).

(5) $$\frac{m_{{\mathrm{TiO}}_{2}}}{m_{{\mathrm{TiO}}_{2}-\mathrm{BCN}}}=\frac{\varepsilon_{{\mathrm{TiO}}_{2}-\mathrm{BCN}}.\;{N_{D}}_{{\mathrm{TiO}}_{2}-\mathrm{BCN}}}{\varepsilon_{{\mathrm{TiO}}_{2}}.\;{N_{D}}_{{\mathrm{TiO}}_{2}}}$$

It can be concluded that the TiO_2_-BCN heterostructure has a greater value of factor $\varepsilon \cdot N_{D}$ than the bare TiO_2_. This can be partially ascribed to a higher $N_{D}$ in the TiO_2_-BCN sample, induced by the plasma treatment during BCN growth. The higher donor density in the TiO_2_-BCN heterostructure is also in good agreement with the increase in the photocurrents in this sample in comparison with TiO_2_ (Figure 6b). Moreover, the dielectric constant of the TiO_2_-BCN heterostructure was also modified as the factor $\frac{\varepsilon_{{\mathrm{TiO}}_{2}-\mathrm{BCN}}.\;{N_{D}}_{{\mathrm{TiO}}_{2}-\mathrm{BCN}}}{\varepsilon_{{\mathrm{TiO}}_{2}}.\;{N_{D}}_{{\mathrm{TiO}}_{2}}}$ is not constant with the frequency. With the value of $\varepsilon_{{\mathrm{TiO}}_{2}}.$ ${N_{D}}_{{\mathrm{TiO}}_{2}}$, the product $\varepsilon_{{\mathrm{TiO}}_{2}-\mathrm{BCN}}.\;{N_{D}}_{{\mathrm{TiO}}_{2}-\mathrm{BCN}}$ vs. the frequency can be obtained, as shown in Figure 7d. This factor also follows an exponential decay tendency, similar to that obtained for TiO_2_ [37] in the region of 100–1000 Hz, ascribed to the $\varepsilon_{{\mathrm{TiO}}_{2}-\mathrm{BCN}}$ as the ${N_{D}}_{{\mathrm{TiO}}_{2}-\mathrm{BCN}}$ is expected to be independent of the frequency.

To summarize our results, the photo-electrochemical response characterizations suggest that the BCN layer acts as an efficient electrocatalyst, improving electron transfer between the electrolyte and the underlying TiO_2_ electrode. It has been reported before that these good electrocatalytic properties are related to the heterogeneity of these compounds, formed by highly doped C-rich and h-BN domains. Substitutional doping and grain boundaries defects act as electrocatalytic sites for OER [15]. The improvement of the electrocatalytic properties of the samples is also confirmed by EIS measurements, that show an increase in the product of the donor density and the dielectric constant induced by BCN growth. On the other hand, it must be noticed that the BCN layer is very thin and absorbs mainly in the UV region [16], so it is not expected that it contributes to the creation of electron–hole pairs due to the appearance of new energy levels

## 4. Conclusions

We have successfully changed the composition and structure of TiS_3_ by thermal annealing at 300 °C to obtain TiO_2_ without changing the nanoribbon morphology of the samples. The XRD and Raman measurements reveal a change from TiS_3_ monoclinic to anatase tetragonal TiO_2_, and the diffuse reflectance measurements a change in the gap from 0.94±0.04 eV to 3.3±0.4 eV. On top of the TiO_2_ nanoribbons, we have successfully grown a BCN layer by plasma-enhanced CVD without affecting the TiO_2_ structure and morphology as it has been proved by XRD, Raman and XPS techniques. The analysis of the chemical composition and bonding scheme of the BCN layer revealed that our layer is composed of C and h-BN nanodomains with high mutual doping levels of B and N in C, and of C in BN, respectively. 

The heterostructure TiO_2_-BCN has been measured in a photoelectrochemical cell, corroborating the good photoelectrocatalytic properties of the BCN to carry out the OER when compared with the bare TiO_2_ substrate. Cycling stability tests and Raman measurements after the experiments demonstrated that the BCN layer remained on the sample and that the structure of the sample has not changed, showing the high stability of our samples. Finally, with EIS measurements, flat band potentials for TiO_2_ and TiO_2_-BCN have been determined to have the same value (0.2±0.1 V vs RHE). By analyzing the slopes of the Mott–Schottky plots, it has been determined that the factor $\varepsilon \cdot N_{D}$ is higher in the case of the TiO_2_-BCN sample which is in good agreement with the increase observed in the photocurrents. In conclusion, the present results point out the excellent photoelectrocatalytic properties of the BCN as a metal-free material to be used in water splitting devices.

## Data Availability

Not applicable.

## Supplementary Materials

The following are available online at https://www.mdpi.com/article/10.3390/ma14195490/s1, Figure S1: Photograph of a sample before and after the oxidation, Figure S2: Scheme of the photoelectrochemical cell used in this work, Figure S3: Raman spectra of one of the samples with BCN. The spectra have been measured in an external nanoribbon (border) and one in the center of the sample, Figure S4: XPS measurement between 384eV and 413 eV. N1s is overlapped with a small signal of the Ta4p_3/2_ peak, ascribed to the tantalum clips used to fix the sample to the sample holder, Figure S5: Photocurrent of TiO_2_-BCN heterostructure under Xe lamp illumination at 0.6 V vs Ag/AgCl in 0.1 M KOH aqueous solution, Figure S6: (a) LSW at different scan rates (b) CV at different scan rates (c) Difference between the anodic and cathodic current of the CV measurements, Figure S7: To prove the stability of our electrode, series of cyclic voltammetry at 0.05mV/s have been done. This figure shows the current at the maximum potential applied (1.8V vs RHE) for each one of the cycles, for the TiO_2_-BCN sample in 1.0M KOH aqueous electrolyte, Figure S8: Raman spectra of TiO_2_ and TiO_2_-BCN before the photoelectrochemical measurements (PEC) and TiO_2_-BCN after the PEC, Figure S9: Donor density of the TiO_2_ sample, Table S1: Position (binding energy, BE), full peak width at a half maximum (FWHM), and relative intensities (with and without oxygen contribution) for the TiO_2_-BCN and bare TiO_2_ samples, Table S2: Comparison between TiO_2_ and TiO_2_-BCN of the current density in dark condition at 1.85V vs RHE (I_dark_), photocurrent at 1.85V vs RHE (I_ph_) and flat band potential, Table S3: Fitting parameters of graphs Figure 7c,d, to an exponential decay: $y=y_{0}+A.\exp\left(-\frac{f}{t}\right)$ where y is the parameter of the Y-axis, and f is the frequency.
