# Supplementary material for: Borocarbonitride Layers on Titanium Dioxide Nanoribbons for Efficient Photoelectrocatalytic Water Splitting
Source: Materials (Basel). 2021 Sep 23;14(19):5490. doi: 10.3390/ma14195490 (PMC8509612; doi:10.3390/ma14195490)
Supplement: Supplementary file 1 [file materials-14-05490-s001.zip › materials-1380768-supplementary.pdf]

Supplementary Materials

Borocarbonitride Layers on Titanium Dioxide Nanoribbons for Efficient Photoelectrocatalytic Water Splitting

Nuria Jiménez-Arévalo [1], Eduardo Flores [2], Alessio Giampietri [3], Marco Sbroscia [3], Maria Grazia Betti [3], Carlo Mariani [3], José R. Ares [1], Isabel J. Ferrer [1,4], Fabrice Leardini [1,4]

[1] Departamento de Física de Materiales, Universidad Autónoma de Madrid, Campus de Cantoblanco, E-28049 Madrid, Spain
[2] Centro de Nanociencias y Nanotecnología (CNyN), Universidad Nacional Autónoma de México (UNAM), Ensenada, Baja California C.P. 22860, Mexico
[3] Dipartimento di Fisica, Università di Roma 'La Sapienza', I-00185, Italy
[4] Instituto Nicolás Cabrera, Universidad Autónoma de Madrid, Campus de Cantoblanco, E-28049 Madrid, Spain

Materials and Methods

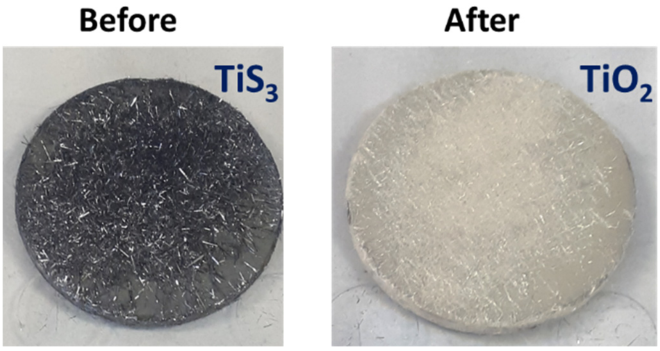

Figure S1. Photograph of a sample before and after the oxidation.

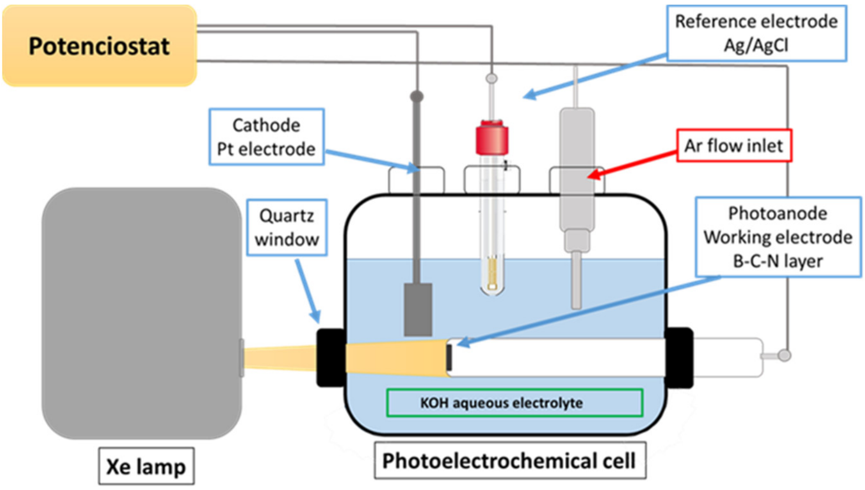

Figure S2. Scheme of the photoelectrochemical cell used in this work.

Results and Discussion

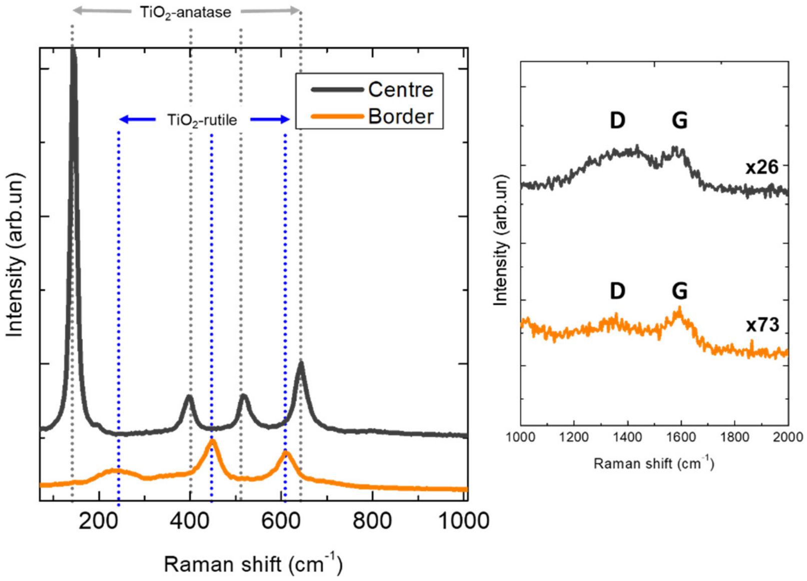

Figure S3. Raman spectra of one of the samples with BCN. The spectra has been measured in an external nanoribbon (border) and one in the center of the sample.

Table S1. Position (binding energy, BE), full peak width at a half maximum (FWHM) , and relative intensities (with and without oxygen contribution) for the TiO2-BCN and bare TiO2 samples.

Sample	Core level	Core level component	BE (eV)	FWHM (eV)	Area	Relative intensity (%)	Relative intensity excluding oxygen (%)
TiO2-BCN	B1s	B-C	189.40	1.21	87.52	12.39	3.85
		B-N	190.87	1.95	156.62	22.17	64.15
		B-O	192.70	2.45	462.40	65.45	
	C1s	C-B	282.91	1.20	25.12	2.29	2.53
		C-C	284.64	1.73	849.17	77.48	85.47
		C-N	285.48	1.56	120.37	10.98	12.10
		C-Ox	286.43	1.52	101.29	9.24	
	Ti2p	Ti3+ (Ti2p$_{3/2}$)	457.55	0.61	9.04	0.45	
		Ti4+ (Ti2p$_{3/2}$)	459.50	1.47	1207.50	60.77	
		Ti4+ (Ti2p$_{1/2}$)	465.15	2.47	770.31	38.77	
TiO2	Ti2p	Ti4+ (Ti2p$_{3/2}$)	459.60	1.39	5378.50	62.31	
		Ti4+ (Ti2p$_{1/2}$)	465.29	2.35	3253.50	37.69	

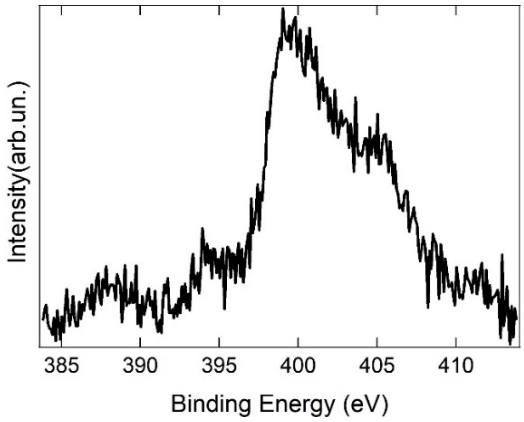

Figure S4. XPS measurement between 384eV and 413 eV. N1s is overlapped with a small signal of the $Ta4p_{3/2}$ peak, ascribed to the tantalum clips used to fix the sample to the sample holder.

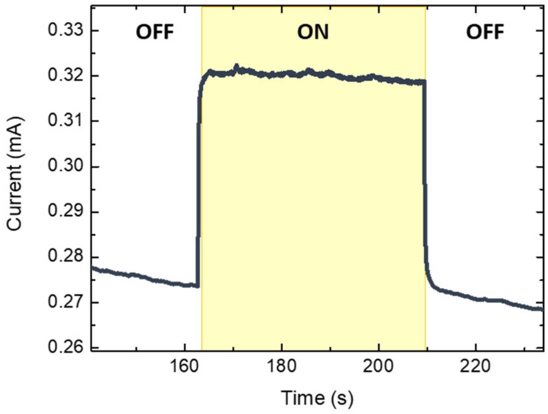

Figure S5. Photocurrent of TiO_2-BCN heterostructure under Xe lamp illumination at 0.6 V vs Ag/AgCl in 0.1 M KOH aqueous solution.

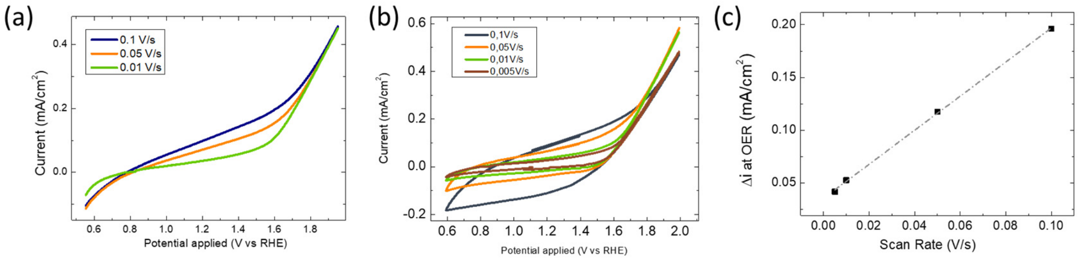

Figure S6. (a) LSW at different scan rates (b) CV at different scan rates (c) Difference between the anodic and cathodic current of the CV measurements.

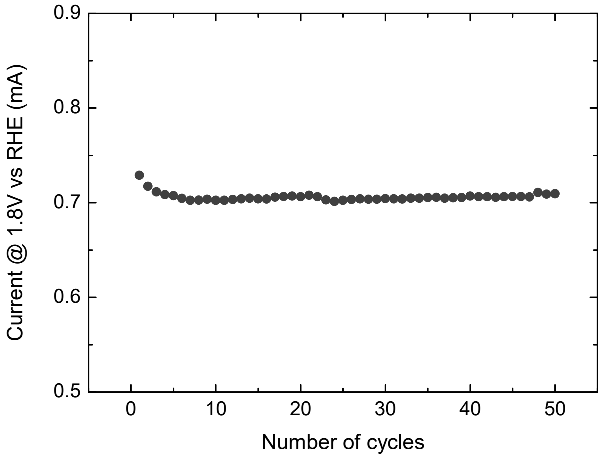

Figure S7. To prove the stability of our electrode, series of cyclic voltammetry at 0.05mV/s have been done. This figure shows the current at the maximum potential applied (1.8V vs RHE) for each one of the cycles, for the TiO_2-BCN sample in 1.0M KOH aqueous electrolyte.

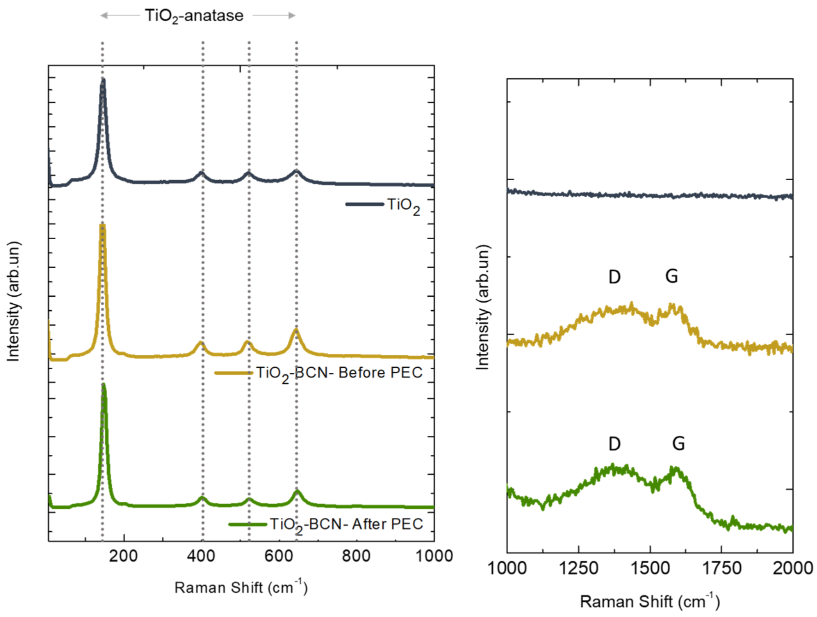

Figure S8. Raman spectra of TiO_2 and TiO_2-BCN before the photoelectrochemical measurements (PEC) and TiO_2-BCN after the PEC.

Table S2. Comparison between TiO_2 and TiO_2-BCN of the current density in dark condition at 1.85V vs RHE (I_{dark}), photocurrent at 1.85V vs RHE (I_{ph}) and flat band potential.

	TiO_2	TiO_2-BCN
I_{dark} (@ 1.85V vs RHE)	0.006 µA/cm^2	0.071 µA/cm^2
I_{ph} (@ 1.85V vs RHE)	1.78 µA	47.6 µA
E_{fb}	0.2 ± 0.1 V vs RHE	0.2 ± 0.1 V vs RHE

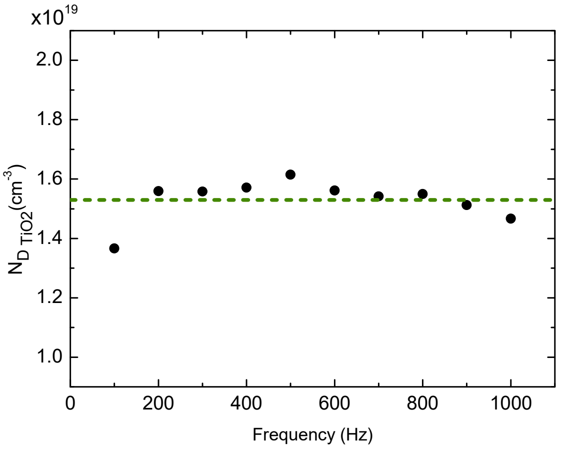

Figure S9. Donor density of the TiO_2 sample.

Table S3. Fitting parameters of graphs Figure 7c and d, to an exponential decay: $y = y_0 + A.\exp\left(-\frac{f}{t}\right)$ where y is the parameter of the Y-axis, and f is the frequency.

	y_0	A	t
$\frac{\varepsilon_{\text{TiO2-BCN}}.N_{D_{\text{TiO2-BCN}}}}{\varepsilon_{\text{TiO2}}.N_{D_{\text{TiO2}}}}$	7.6 ± 0.6	133 ± 2	236 ± 6
$\varepsilon_{TiO2-BCN}.N_{DTiO2-BCN}$	$(3.0 \pm 0.5) \cdot 10^{22}$	$(9.3 \pm 0.4) \cdot 10^{23}$	$(1.5 \pm 0.2) \cdot 10^{2}$
